# Muscle myography for human–machine interfaces: a review of sensing modalities and control interfaces

**DOI:** 10.3389/fbioe.2026.1759542

**Published:** 2026-05-15

**Authors:** Sudhir Solomon Zhuwawu, Mahonri Owen, Albert Bifet, Anany Dwivedi

**Affiliations:** 1 AI Institute, School of Computing, University of Waikato, Hamilton, New Zealand; 2 Wearables Lab, School of Engineering, University of Waikato, Hamilton, New Zealand

**Keywords:** human-computer interaction (HCI), human-machine interface (HMI), machine learning, muscle myography, muscle-machine Interface (MMI), prosthetics, rehabilitation

## Abstract

Muscle myography encompasses a family of techniques for sensing muscle activity through its electrical, mechanical, and optical manifestations, offering non-invasive and embodied pathways for human–machine interaction. Unlike traditional input devices that are hand-centric and device-bound, myography-based interfaces enable hands-free, inclusive control for prosthetics, exoskeletons, teleoperation, and immersive computing, while also opening opportunities in rehabilitation and health monitoring. This review surveys the state of muscle myography with an emphasis on its implications for human–machine interfaces (HMIs). We examine established and emerging modalities—including electromyography (EMG), mechanomyography (MMG), forcemyography (FMG), electrical impedance myography (EIM), optomyography (OMG), and the recently introduced lightmyography (LMG)—highlighting their respective strengths, weaknesses, and the trade-offs between signal quality, robustness, and wearability. We also identify recurring challenges such as limb position effects, motion artifacts, sensor reliability, and clinical acceptability, and discuss strategies such as hybrid sensing, sensor fusion, and machine learning aimed at mitigating these issues. For the HMI community, the significance of muscle myography lies not only in technical performance but also in its potential to redefine interaction design, shifting toward interfaces that operate less as external devices and more as natural extensions of the human body.

## 1 Introduction

The field of HMI has witnessed significant breakthroughs in the past decade. Most recently, researchers at the University of California were able to decode speech from brain activity using a minimally invasive Brain-Computer Interface (BCI) (Littlejohn et al., 2025). While this achievement is remarkable and has far-reaching implications, its applications will be limited due to its medically invasive nature. Non-invasive encephalography-based BCIs (EEG) have also produced promising results in the fields of prosthetic control (Roy et al., 2018; Hong et al., 2020; Müller-Putz et al., 2005; Bright et al., 2016), exoskeleton control (Kilicarslan et al., 2013), and computer input (Taha Beyrouthy et al., 2017). However, EEG signals are vulnerable to interference, are affected by motion artifacts, and often require high levels of user attention to maintain strong signals (Light et al., 2010; Kilicarslan et al., 2013). In addition, phenomena such as BCI illiteracy (Maskeliunas et al., 2016) limit the viability of consumer-grade non-invasive EEG devices. As an alternative, Muscle-Machine Interfaces (MMIs) that leverage muscle myography represent one of the most crucial areas of HMI. These devices will unlock alternative ways to interact with assistive devices, machines, computers, and the ever-growing virtual world around us. For example, MMIs can provide greater functionality, such as controlling a supernumerary arm in real time or interacting with a virtual environment, while performing other activities concurrently (Gnocco et al., 2023). Muscle myography-based MMIs enable hands-free control by decoding muscle activity, diverting the control task to other body sites (Godoy et al., 2022a). This allows users to control or interact with devices without using their hands. Similarly, people with disabilities can leverage these intuitive control schemes to interact with assistive devices, computers, and prostheses easily. Some of the key areas where muscle myography-based MMIs are being applied include assistive applications such as prostheses control, exoskeleton control, and teleoperation, in addition to computer interface and virtual/augmented reality.

Current robotic prosthetic hands possess similar levels of dexterity to the human hand, but prosthetic control has been hindered by the lack of intuitive control methods that can effectively translate user intent into seamless control actions. Preferred methods, such as agonist/antagonistic myoelectric control, offer a cumbersome experience that prevents users from exploiting the full functionality and often results in abandonment of these devices (Smail et al., 2021; Yamamoto et al., 2019; Biddiss and Chau, 2007). Studies on the advancements of prosthetics over the past decade (2012/13 to 2022/23) have found that the impact on the abandonment rates has not been significant, despite research breakthroughs in control and feedback (Salminger et al., 2022; Dey et al., 2023). This was attributed to factors such as difficulties in implementing proposed solutions in the real world, due to research conducted in constrained laboratory settings and designs that lack practical user considerations, in addition to a majority of research using only healthy participants (Dey et al., 2023). Currently, the trend of incorporating machine learning to detect and predict muscle activations based on patterns in signals from muscle myography sensors has yielded encouraging results. This machine learning-driven approach promises to unlock greater functionality and seamless integration of prosthetic hands into daily life activities. However, many limitations still hinder the transition from lab to clinical or daily life applications. These include factors such as data collection in highly constrained lab environments that do not represent real-world scenarios and physiological factors such as muscle fatigue and sweat that alter the nature of the signals.

Robotic exoskeletons have gained massive traction in recent years, drawing more research and commercial interest. Their applications include rehabilitation, strength enhancement for healthy individuals or those with special needs, improved mobility for individuals with disabilities, and industrial and military applications. However, to fully exploit the benefits of exoskeletons, intuitive control methods are required. Muscle myography-based MMIs can meet this requirement through hands-free interaction, causing minimal cognitive burden. In addition, exoskeletons using muscle myography-based MMIs can detect and respond to operator fatigue (Net’uková et al., 2022).

Muscle myography-based MMIs have significant potential for enabling intuitive teleoperation. This application often necessitates the control of multiple degrees of freedom. Using traditional HMIs such as joysticks and touchscreens has the limitation of completely occupying the user’s hands. Other approaches have included foot control (Huang et al., 2021b; Huang et al., 2021a), exoskeletons for motion capture (Jo et al., 2013), and EEG (Qiu et al., 2016). With MMIs, the user can intuitively control the robot whilst simultaneously using their hands for other tasks. Examples include controlling a supernumerary arm during rehabilitation (Gnocco et al., 2023) or daily life for people with disabilities (Fall et al., 2016), robotic surgery (replacing traditional hand and foot controllers) (Nguyen et al., 2023), or assembly tasks. In this way, muscle myography-based MMIs could provide intuitive and low-cognitive-taxing control schemes to enhance user control and functionality.

Typical computer interfaces, such as keyboards, mice, and touchscreens, are difficult to use for people with disabilities such as limb loss, strokes, neurological disorders, and others. In such cases, alternative interface methods are required. Some popular approaches include decoding of EEG signals, tongue-controlled devices (Kirtas et al., 2021; Sapaico et al., 2011; Park et al., 2011), gaze detection, and voice control. The use of muscle myography-based MMIs is also a popular approach. Minor muscle activations can be mapped to computer input with low cognitive load, and noise and interference problems that affect EEG and voice control can be avoided.

Virtual and Augmented Reality (VR and AR) can also benefit from muscle myography-based MMIs. These can be used to replace the obtrusive motion tracking systems and input devices commonly used with VR and AR. By using pattern recognition techniques, signals related to real-time muscle activity, such as hand gestures and motion, can be decoded and used to interact with the virtual space with minimal equipment requirements (Zhang et al., 2022). This has major implications for rehabilitation, where virtual environments can be used to provide rehabilitation with greater accessibility and immersive experiences (Munoz-Novoa et al., 2025). In addition, the continued growth of virtual environments will increase the need for more intuitive control interfaces for both able-bodied users and users with disabilities.

This paper provides a narrative review of muscle myography-based MMIs in HMI, encompassing electromyography (EMG), mechanomyography (MMG), force myography (FMG), electrical impedance myography (EIM), optomyography (OMG), and lightmyography (LMG). Each technique measures some physical parameter produced by muscle contractions. Techniques such as surface EMG, FMG, and EIM measure superficial parameters relating to muscle activity near the skin surface, while MMG can detect muscle activity from deeper muscles, and OMG and LMG use light that penetrates beneath the skin surface. Additionally, we explore hybrid approaches that combine two or more muscle myography techniques. Hybrid approaches attempt to leverage the strengths of the combined techniques, resulting in composite data that captures more information about muscle activity (Sanford et al., 2017). While muscle myography-based MMIs are promising, there remain numerous challenges limiting their adoption. This paper also examines the key challenges and current approaches to addressing them. At the time of writing this paper, there were few review papers on multi-modal muscle myography and its applications to MMI, and none covering newer techniques, such as OMG and LMG. However, there are notable review papers for the various individual muscle myography techniques, such as EMG (Merletti et al., 2010; Jarque-Bou et al., 2021; Samuel et al., 2019, FMG Xiao and Menon, 2019; Sherif et al., 2024, MMG Beck et al., 2005a; Dillon et al., 2011, and EIM Sanchez et al., 2021; Youssef Baby et al., 2024).

The remainder of this paper is organized as follows. Section 2 presents the methods used to select the studies included in this paper. Section 3 describes the signals produced by muscle activity and how they propagate through the various layers. Section 3 also explores existing muscle myography techniques, describing how they work, their strengths, and weaknesses. Section 4 presents cross-modality comparisons and best use cases for each technique. Furthermore, Section 5 examines the various applications of muscle myography in current studies. In Section 6, the challenges that limit the real-world performance of muscle myography-based systems are discussed. Finally, Section 7 summarizes our review and provides a brief outlook on the prospects of muscle myography-based MMIs.

## 2 Methodology

This section outlines the methods used to select the studies included in this review. While the review is narrative in nature, the selection of studies was conducted systematically to ensure a representative description of the current state of each muscle myography technique. The studies were chosen based on the following selection criteria.

- Databases - only papers from trusted databases were included in this review. The specific databases used were IEEE, PubMed, Scopus, and Web of Science.
- Contextual Relevance - this review is aimed at applications in HMI; therefore, only papers that present findings relevant to HMI applications were considered. Studies on rehabilitation and medical applications whose findings could be applied to augment HMIs for user health monitoring were also considered.
- Research Contribution - each paper was assessed for its contribution to the relevant techniques. Contributions considered included novelty of design/implementation, signal processing, signal decoding (machine and deep learning), control action mapping, user studies, real-world testing, and detailed exploratory studies that reveal the underlying principles.

Studies that met the contextual relevance criterion were evaluated based on the title and abstract. Upon meeting this criterion, the research contribution was assessed by full-text screening. In cases where researchers used similar methods with similar results, preference was given based on chronological order. In cases of conflicting results, a thorough analysis was conducted to understand the various methodological approaches and findings in the context of the existing research landscape.

The review aimed to be inclusive, considering studies that followed established and emerging research practices, to gain a comprehensive perspective on the topic. An iterative, multi-step search approach was adopted, involving repeated cycles of literature searching, screening, and analysis to progressively refine and expand the set of studies included in this review. The review was limited to only studies published in English or that had readily available English translations within the specified databases. No specific time restrictions were applied, and the search cycle ended in March 2026.

**FIGURE 1:**
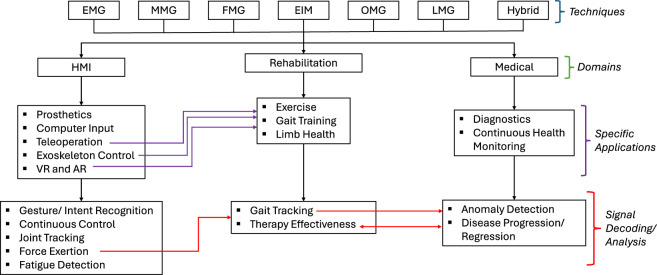
Literature search tree.

Figure 1 summarizes the literature search process. The search terms used in this review can be synthesized by traversing the tree from *Technique* → *Domain* → *Specific Application* → *Signal Decoding/Analysis*, and then modified to ensure grammatical correctness. This process effectively summarizes the search terms used; however, it is not exhaustive. The search terms were adjusted for best performance on the different databases, and some specialized studies were identified individually. This review is not intended to be exhaustive, but rather aims to provide a comprehensive narrative of the broad landscape of muscle myography-based MMIs, discussing their working principles, strengths, weaknesses, obstacles, and opportunities within the context of human-machine interaction.

## 3 Existing muscle myography techniques

In this section, we first describe the principles for acquiring muscle myography signals, followed by a description of EMG, MMG, FMG, EIM, OMG, LMG, and finally the hybrid approach. More precisely, individual techniques are discussed, describing how they work, traditional and current approaches, and practical considerations such as optimizing sensor location and number, as well as the acquisition and processing of sensor signals. We also discuss their strengths and weaknesses. The general challenges facing all muscle myography techniques are discussed in Section 6.

### 3.1 Detection principles of muscle myography

**FIGURE 2:**
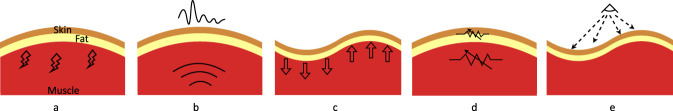
**(a)** Electrical activity from muscles **(b)** Vibration from muscle activity propagating to the skin’s surface **(c)** Pressure waves produced by volumetric changes of the muscles, **(d)** Changes in electrical impedance of muscles, skin, and fat layers due to deformation **(e)** Skin, fat layer, and muscle deflections.

Muscle activations give rise to several physiological processes and their associated physical manifestations, which can be detected by different sensing modalities, as depicted in Figure 2. Each muscle myography technique targets a specific event(s) by measuring the associated physical parameters. The most prevalent physical parameters are the changes in the length and cross-sections of the muscles, i.e., the volumetric changes and the stiffness (Xiao and Menon, 2019; Islam et al., 2020; Delva and Menon, 2017). These volumetric changes result in the propagation of pressure waves or lateral forces along the active muscles and across the skin surface, also resulting in deformation of the skin and subcutaneous layers in various directions. In addition, the volumetric changes also result in a change in the electrical impedance of the muscles and changes in the skin and fat layer thicknesses, due to stretching and contracting, which also alter their electrical impedance (Briko et al., 2021a).

During the initial activation of the muscle and the subsequent relaxation, vibrations propagate through the muscles, surrounding tissues, skin layers, and fluid (Silva et al., 2004). The vibrations are produced by the movements of the muscles and the resulting volumetric changes, in addition to lateral oscillations of the muscles (Beck et al., 2005a; Antonelli et al., 2009). These vibrations are largely transient and are more prominent during the transition from relaxed-contracted or *vice versa* (Beck et al., 2005a). In the middle (for example, during a grasp), there are still vibrations, but these are less observable and are more affected by noise. It should also be noted that the vibrations from the activations of multiple muscles result in composite vibrations that can be measured away from the originating source (Silva et al., 2004). Consequently, muscle myography techniques that measure these vibrations can detect the activation of superficial and deeper muscles. Due to the nature of the propagation of the vibrations, effects of damping must be taken into account when recording these signals (Silva et al., 2004).

Currently, the most favored parameter to observe is the electrical activity of the muscles themselves (Sul et al., 2025). This technique forms the basis for EMG, which is the most common technique to both observe and assess muscle activity in MMIs and medical applications such as diagnostics and rehabilitation. Muscle activity is initiated by motor neurons that relay commands from the brain to the muscles. When the muscle is activated, electrical signals that propagate along the muscle fibers are generated and can be measured either at the skin’s surface or directly from the muscles (De Luca, 2007). These signals provide rich information that, if successfully decoded, can provide the most clarity on muscle activations.

### 3.2 Electromyography (EMG)

Most EMG-based MMIs refer to its surface variant (sEMG), where the electrical signals are measured at the skin’s surface. Consequently, a significant portion of this information-rich signal is distorted by factors such as impedance (muscle, skin, fat layers, internal fluids) in addition to surface conditions of the skin itself (dryness, sweat, scars, or abrasions) which are unpredictable (Merletti et al., 2010). Additionally, the signal is also significantly affected by motion artifacts. These effects can be bypassed by measuring the signal directly from the muscle fibers using invasive techniques such as needles (Rubin, 2019; Merletti and Parker, 2004). In doing so, the information-rich signals can be mostly preserved. However, this invasive approach is not feasible in wearable MMIs or multi-use cases; it is mostly used in medical domains such as diagnostics, but has also been used in prosthetic control (Smith et al., 2014). Other factors, such as damage to the neuromuscular pathways due to physical trauma or disease are also limiting factors for EMG (Roy et al., 2018).

Despite these drawbacks, sEMG remains largely popular as the signal still retains a wealth of useful information that can be decoded using feature extraction algorithms and then converted by classifier algorithms into inputs for various applications. One of the major advantages of EMG is its ability to decode or predict user intentions from muscle activations using pattern recognition methods (Tian et al., 2023; Pancholi et al., 2022; Ghildiyal et al., 2022; Tushir et al., 2024; Simon et al., 2024; Mhiriz et al., 2024; Kok et al., 2024; Williams et al., 2024; Camargo and Young, 2019; Vasan and Pilarski, 2017; Benitez Lopez et al., 2019). These highlighted works successfully decoded sEMG signals to predict the intended grasping action of the subjects, and used this to control prosthetic hands. EMG signals were recorded as subjects performed gestures or imagined performing gestures in the case of amputees. ML models were then trained to decode user intentions based on the predicted gesture and then initiate the corresponding action in the prosthetic hand. These works demonstrate how EMG-based intent recognition combined with autonomous control can significantly enhance prosthetic control.

We also found that one of the reasons for the popularity of sEMG is the availability of open-access EMG signal datasets, such as in Atzori et al. (2014), Atzori et al. (2012); Ozdemir et al. (2022); Kaczmarek et al. (2019); Phinyomark and Scheme (2018). These datasets are very important, allowing researchers to explore various deep learning techniques, pre-processing, feature extraction, new perspectives, and uses of EMG data, such as emotion recognition Phinyomark and Scheme (2018). While these datasets are valuable tools for researchers, the data is collected from highly constrained environments to reduce the noise. Additionally, electrode placement is typically performed by a physician in case of traditional sEMG, such as in Ozdemir et al. (2022). Consequently, this data does not represent the highly dynamic and often unpredictable real-world scenarios where these devices will be used Fougner et al. (2011); Geng et al. (2012); Campbell et al. (2020). This means that any results based solely on these datasets will not necessarily translate into real-world performance. In fact, Jiang et al. (2013); Simon et al. (2024) found that the correlation between offline and online performances is not strong; therefore, online performance is a more important metric. Furthermore, physiological factors such as fatigue and phenomena such as the limb position effect (discussed in Section 6.2) will significantly degrade the performance of devices trained on this data when used in the real world.

#### 3.2.1 Sensor placement

The location of EMG sensors (especially sEMG) is hugely important. Poor placement can result in cross-talk, where two or more sensors record the activity of the same muscle instead of their respective target muscles (Merletti et al., 2010). Optimal placement of the sensors directly on the active muscles will result in higher clarity signals. Consequently, sEMG is particularly sensitive to sensor shifting and lift-off, significantly degrading the performance of classifiers. Therefore, placing the electrodes at optimal locations and spacing is critical. Additionally, ensuring good electrode adhesion and electrical contact is required. This often requires preparation of the skin at the target location in addition to application of some conductive agent, such as gels or conductive pastes (Merletti and Cerone, 2020; Daley et al., 2012). In consumer-grade devices, this is avoided by simply securing the electrodes in an armband that applies pressure. However, this means these devices tend to be noisy due to sensor shift and skin conditions such as sweating or dryness.

#### 3.2.2 High density approach

**FIGURE 3:**
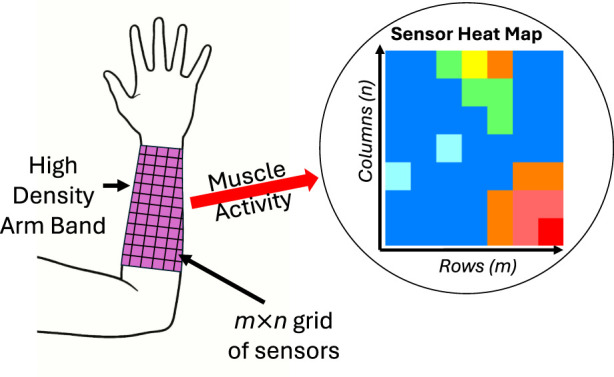
Generic representation of the high-density approach. This setup forms a 2D display of muscle activity.

Clinical acceptability of EMG devices requires that the number of electrodes are limited to 8 or less (Huang et al., 2008; Daley et al., 2012). Fewer electrodes also means less computational resources needed to record and process the signals (Stango et al., 2014). However, increasing the number of electrodes increases the density of information that can be recorded, leading to higher classification accuracies up to a point before plateauing (Zhou et al., 2007; Parker et al., 2006). Simon et al. (2024) found that doubling the number of electrodes from 8 to 16 significantly improved the control in healthy participants. Naturally, the high-density approach has been the subject of much research, with the main concept consisting of a 2D array of EMG electrodes as shown in Figure 3. This arrangement forms a display or map of electrical activity. Daley et al. (2012) used up to 64 EMG channels with 8 × 8 evenly spaced electrodes for healthy limbs. When applied to amputees, the number of channels had to be reduced (up to 32). This approach was also used as an assessment method to find the best location for electrode placement by identifying the ‘hot spots’ in the EMG heat maps. They also found that the number of tasks that could be accurately classified was significantly less for amputees (4–6) than for healthy subjects (10–13). Stango et al. (2014) also explored the high-density approach to mitigate electrode shift and malfunctioning channels, creating a more robust EMG system for prosthetic control. However, a significant portion of the EMG channels remained mostly dormant during the testing of different gestures. Pan et al. (2015) also used the high-density approach, with 192 electrodes, to improve the robustness of EMG against sensor shift. The implications are that HD-EMG can mitigate sensor shift, which is highly detrimental in EMG; however, this comes at the cost of clinical acceptability and increased complexity.

#### 3.2.3 Signal conditioning

The goal of signal conditioning is to convert the signal into a form suitable for data acquisition. This includes signal amplification, filtering, and noise rejection (Merletti and Cerone, 2020). The signal needs to be amplified to match the input voltage range for any connected instruments, such as analog-to-digital converters. Before amplification, the magnitude of the signal is very small and therefore susceptible to interference such as power line interference (Sul et al., 2025; Galiana-Merino et al., 2013). Filtering the signal eliminates the presence of unwanted harmonics outside the EMG signal’s frequency band. Noise and interference within the frequency band also need to be filtered or rejected. We recommend the paper by Merletti and Cerone (2020) for an in-depth study and tutorial on the detection, conditioning, and preprocessing of sEMG signals.

#### 3.2.4 Feature extraction and classification

EMG techniques have greatly benefited from the development of feature extraction and classification algorithms, capable of characterizing the chaotic signals to identify discernible patterns. The choice of which features to extract directly affects the subsequent performance of the classifiers. Time-domain (TD) features such as the root mean square (RMS) or the mean absolute value (MAV) are extracted directly from the preprocessed or raw signals and are hence easier to compute and apply in real-time (Phinyomark et al., 2012; Sid’El Moctar et al., 2023; Too et al., 2017; Turner et al., 2021). Frequency-domain (FD) features are extracted from the signal transformed into the frequency domain. These features are useful for classifying movements and fatigue (Too et al., 2017) and may be robust to noise when compared with TD (Sevim, 2022). Time-Frequency Domain (TFD) features combine the time and frequency domains, providing better insight into the muscle activity (Karheily et al., 2022).

Once the desired features are extracted, the next stage is to classify the signals to predict/recognize classes of gestures, motion, fatigue, force, and others. Various classifiers have been implemented from lightweight algorithms such as simple artificial neural networks (ANNs) (Tushir et al., 2024) to heavyweight deep learning techniques such as temporal multi-channel vision transformers (Godoy et al., 2022c).

### 3.3 Mechanomyography (MMG)

MMG measures the dynamic action of muscles through the vibrations produced. These vibrations are known as ‘muscle sounds’ (Orizio, 1993; Silva et al., 2004) and MMG is also referred to by other names such as soundmyography, phonomyography, acousticmyography, and vibromygraphy (Orizio, 1993). Sensors that can detect these vibrations at the skin’s surface include condenser microphones (Fara et al., 2013; Alves and Chau, 2010b; Orizio, 1993), piezo-plates (Asheghabadi et al., 2021), piezoelectric film (Pan et al., 2020), laser distance sensors (Dillon et al., 2011), and accelerometers (Silva et al., 2004; Shima and Tsuji, 2009) as the most common options. However, combinations of these sensors are also used. For example, Silva et al. (2004) used three pairs of accelerometers and microphones placed at the stump. While Asheghabadi et al. (2021) used piezo-plates to measure the vibrations and strain gauges to capture the acoustic features. The propagation of the vibrations until they reach the skin means that MMG signals can be measured from any location near the active muscles, and direct contact is not required (Silva et al., 2004). This also means that effects of damping and sensor fixation have to be carefully considered (Antonelli et al., 2009). Castillo et al. (2020) identified the skin-sensor contact area as a critical source of damping and therefore developed sensors that could change the normal surface pressure of the skin-sensor contact area, resulting in higher conductivity of the vibrations.

MMG is often presented as a more robust alternative to EMG. It is less affected by electrical interference, not limited to superficial muscles, more tolerant to sensor shift and placement, and has a higher signal-to-noise ratio (SNR) (Pan et al., 2020; De la Rosa et al., 2010; Orizio et al., 2003, Orizio et al. 1996). Therefore, MMG is considered more suitable for long-use cases, such as prosthetic control (De la Rosa et al., 2010; Castillo et al., 2020) and BMI applications (Fara et al., 2013). However, MMG is not immune to crosstalk. Dynamic coupling of the muscles due to the sensor fixation can result in crosstalk (Orizio, 1993). MMG is also more affected by motion artifacts and external vibrations. Additionally, MMG signals are often lower bandwidth compared to EMG, meaning that EMG has more potential applications.

#### 3.3.1 Sensor placement

Sensor placement in MMG must consider the effect of damping above all. Damping is caused by the different layers through which the vibrations are propagating. Therefore, factors such as hydration, normal pressure at the skin-sensor contact area, muscle tightness, thickness of the skin and fat layers, and sensor spacing must be considered carefully (Orizio, 1993; Beck et al., 2005a; Castillo et al., 2020). In addition, skin conditions such as scars or cracking will also affect the signal. Poor sensor mechanical fixation will lead to a significant loss of information (Antonelli et al., 2009). Though sensor placement in MMG is less critical compared to EMG, the location of the sensors may affect the signal (Dillon et al., 2011).

#### 3.3.2 High density approach

As mentioned previously, MMG is highly sensitive to mechanical damping. Adding too many sensors will result in higher damping due to the mass of the sensors and/or the sensor fixation. Consequently, there are no high density approaches. Additionally, because of the way the vibrations propagate, fewer sensors are needed. Asheghabadi et al. (2021) classified 7 finger motions using only one MMG sensor. However, many multichannel designs have been proposed. Alves and Chau (2010b) used up to 6 MMG channels, composed of microphones, each targeting a specific muscle in the forearm. Using linear discriminant analysis, they were able to classify 7 hand movements at 90% accuracy with healthy limbs and 5 contractions at 98% accuracy on an amputee.

#### 3.3.3 Signal conditioning

The vibrations produced by muscle activity are weak; therefore, the signal needs amplification to be usable with any connected instruments/components (De la Rosa et al., 2010). Since MMG is less affected by electrical interference, less filtering of the signal is required. However, low and high-frequency vibrations outside the frequency range of active muscles need to be filtered out. This is often done using a combination of low-pass and high-pass filters (Islam et al., 2013). Noise from other sources, such as motion artifacts, magnetic interference (depending on the type of sensor used), and external sources of vibration, for example, haptic feedback devices, also need to be filtered out.

#### 3.3.4 Feature extraction and classification

Unlike EMG, MMG research has seen less focus on the classification of MMG signals. This may be due to few large public datasets for training and testing, though there has been a growing trend of creating these public datasets such as in Abbas et al. (2025). As a result, simpler classifications have been performed, such as identifying different levels of muscle activation intensity/contraction levels (Fara et al., 2013; De la Rosa et al., 2010; Silva et al., 2004). In Asheghabadi et al. (2021), 7 finger motions were classified using simple artificial neural networks (ANN). Zhang and Xia (2020) used support vector machines (SVM) to classify 3 upper limb motions and 3 forces (9 combinations) to evaluate the progress of rehabilitation. Time, frequency, and time-frequency domain analyses have been used to extract features from the MMG signals (Beck et al., 2005b; Alves and Chau, 2010a), with time-frequency domain producing the best results in comparative studies (Dillon et al., 2011).

### 3.4 Forcemyography (FMG)

FMG measures the pressure or deformation at the skin’s surface, produced by the volumetric changes during muscle activity. FMG sensors detect the surface forces and deformation produced. It follows that force sensing resistors (FSR) are the most popular choice of sensor (Xiao et al., 2014; Vonsevych et al., 2017; Xiao and Menon, 2017; Radmand et al., 2016; Belyea et al., 2019; Delva and Menon, 2017; Chapman et al., 2021). Piezoelectric sensors are another common approach (Centracchio, 2022; Li et al., 2016; Esposito et al., 2018). FMG sensors based on the deformation of optical fibers were proposed and tested in Wu et al. (2020); Fujwara et al. (2018); Fajardo et al. (2019), Fajardo et al. (2024). By measuring the changes in light intensity due to the deformation of the optical fibers, muscle movements could be detected. One of the major advantages of FMG is that the sensors are not affected by skin conditions such as pigmentation, moisture content, and impedance (Fajardo et al., 2024), making it highly desirable in wearable applications. In addition, FMG devices are relatively compact, with low power requirements, and when textile-based sensors are used, they can be potentially integrated into clothing (Lukowicz et al., 2006). In rehabilitation applications, it can be used to assess muscle stiffness (Xiao and Menon, 2019). Other advantages of FMG include lower electromechanical delay in producing sensor responses when compared to EMG Esposito et al. (2020), Esposito et al. (2011). However, FMG is also susceptible to effects of the position of the limb and differences in anatomical parameters, such as muscle tone, which means that sensor calibration is necessary (for example, setting the base pressure) (Ha et al., 2019; Radmand et al., 2016). Another consideration when using FMG is the occurrence of sensor drift (Esposito et al., 2018). Drift in FMG is often produced by mechanical stress, hysteresis, and temperature, which can be addressed by signal conditioning, sensor design, and sensor fixation.

#### 3.4.1 Sensor placement

Sensor placement and muscle selection are crucial in low-channel FMG devices (Ha et al., 2019). Poor sensor spacing will simply result in crosstalk; however, some sensor locations can enable additional information to be captured from other movements. For example, Beck et al. (2005a) were able to detect elbow movements in addition to the local muscle activity since the pressure waves propagated to the sensors near the elbow. Another key consideration to be made is the fitting pressure, the pressure or force used to affix the sensors. If the fitting pressure is too high, low-amplitude muscle activity will not be detected, and dynamic coupling of adjacent muscles can occur, leading to crosstalk. In contrast, low fitting pressure can increase drift.

#### 3.4.2 High density approach

FMG is highly suitable for high-density approaches (HD-FMG). It does not have the same clinical restrictions as EMG or physical restrictions as MMG. In Belyea et al. (2019), an array of 14 × 24 FSR sensors was tested. Their findings showed that this FMG approach could outperform EMG on a number of tested parameters. In addition, they also implemented their design in both sequential and simultaneous regression control. Radmand et al. (2016) also implemented a high-density FSR FMG armband with 14 × 9 sensors. They could classify eight wrist hand motions with this setup and their research also found that HD-FMG could outperform EMG in classifying wrist and hand gestures. However, creating such a large array of sensors means adding complexity and computational load to collect and process the signals, losing some of the inherent advantages of FMG (Rehman et al., 2023).

#### 3.4.3 Signal conditioning

When FSR sensors are used, the raw sensor readings can be classified directly without any signal conditioning. In these cases, the sensor signal is simply taken as the voltage difference in a potentiometer setup (Xiao et al., 2014; Xiao and Menon, 2017, Xiao and Menon, 2019). HD-FMG can also be implemented using only the raw readings (Radmand et al., 2016; Belyea et al., 2019). This greatly reduces the sensor lag; however, amplification and noise filtering, to remove motion artifacts and ensure a constant voltage may be desired even with FSR sensors (Esposito et al., 2018; Islam et al., 2020). Other sensor choices, such as the fiber optic FMG sensors in Wu et al. (2020); Fujwara et al. (2018); Fajardo et al. (2019), Fajardo et al. (2024) and piezoelectric-based sensors, require amplification and noise filtering.

#### 3.4.4 Feature extraction and classification

Since the FMG signals require little to no signal conditioning, the most common features used are the amplitude, RMS values, and gradient, especially in applications using FSR sensors (Xiao and Menon, 2017). Similar to MMG classification, FMG classification has mainly been done using lightweight classifiers such as ANN, SVM, LDA, and decision trees. Even HD-FMG research has also been limited to SVM and LDA classification. This may be due to similar reasons, such as a lack of large datasets. However, due to the simplicity of FMG setups, real-time classification and control have been explored. Li et al. (2016) used extreme machine learning (ELM) to classify 3 arm positions from 8 FSR sensors. Radmand et al. (2016) used an HD-FMG device to classify 8 wrist motions. They also tested the effect of limb position on the real-time classification using SVM and LDA. Their findings suggest that HD-FMG is less affected by the limb position effect compared to EMG. However, the limb position effect still degraded the classifier’s performance.

### 3.5 Electrical impedance myography (EIM)

EIM measures changes in electrical impedance that occur from muscle activity or changes in muscle structure by applying low-amplitude, high-frequency electrical current at the skin’s surface. The measured parameters are the amplitude of the resulting voltage and its phase shift (Rutkove, 2009). It has its origins in medical applications for diagnosing neuromuscular disorders (Ogunnika et al., 2010; Rutkove, 2009; Sanchez and Rutkove, 2017a; Youssef Baby et al., 2024; Sanchez and Rutkove, 2017b), which cause changes to the normal muscle structures. When applied as a diagnostic tool, EIM has the main advantage of requiring minimal subject cooperation (Sanchez and Rutkove, 2017a). Since muscle activity produces volumetric changes that affect local muscle, skin, and fatty layer impedance, EIM can be used to detect and measure muscle activity in MMI applications (Ngo et al., 2022). Xu et al. (2023) used EIM to predict the hand grip force using long short-term memory (LSTM). Cho et al. (2020) used EIM to classify 7 hand patterns using SVM for prosthetic hand control. EIM is less susceptible to electromagnetic interference compared to EMG and can be used to complement its effectiveness (Briko et al., 2021a; Ngo et al., 2022). Other advantages of EIM in MMIs include the ability to easily integrate the electrodes into wearable interfaces or the socket of prosthetic hands. Additionally, it could be used as a health monitoring tool to assess the effectiveness of rehabilitation or early detection of muscle-related health problems (Rutkove, 2009). However, the use of EIM in MMI applications is limited by the complexity of hardware required to accurately measure and interpret the EIM signals (Ngo et al., 2022). Additionally, conditions that alter electrical impedance, such as sweat, hydration, skin, and fatty layer thickness will affect the EIM signal. Similar to EMG, EIM is also affected by motion artifacts and the limb position effect (Cho et al., 2020).

#### 3.5.1 Sensor placement

The most common setup for EIM is the 4-electrode (tetrapolar) setup. Two outer electrodes are used to inject the current, and two inner electrodes are used to measure the voltage. The electrodes should be placed directly above the muscle of interest and along its length. In some medical approaches, the current is injected using electrodes in both hands, while the voltage electrodes are placed over the muscle of interest (Rutkove, 2009). Other important considerations include the selection of the electrode material and their spacing (Pîslaru-Dănescu et al., 2022).

#### 3.5.2 High density approach

Due to the nature of EIM, there are no high-density approaches. Creating too many channels would simply result in the current waveforms interacting. In addition, the amount of current being injected would increase, posing safety concerns. However, multichannel 4-electrode approaches have been proposed for MMI applications. Cho et al. (2020) used a 4-channel setup to measure muscle activity in the forearm for the classification of hand patterns/gestures. In Xu et al. (2023) and Makeeva et al. (2025), dual channels in the forearm were used to classify gestures of the upper limb based on agonist/antagonist muscles.

#### 3.5.3 Signal conditioning

Due to the high frequencies used in EIM, it is less affected by environmental noise meaning that it does not require as much filtering as EMG (Cho et al., 2020). As a result, the lag is much less than with EMG. The main signal conditioning step is amplification, since the injected current is of very low amplitude (Cho et al., 2020). Band-pass filtering to remove low and high frequency noise outside the probing frequency range is also required (Pîslaru-Dănescu et al., 2022).

#### 3.5.4 Feature extraction and classification

EIM is one of the least explored approaches for muscle myography-based MMIs. As a result, there is little work on feature extraction and classification of EIM signals. EIM features are normally extracted in the frequency domain, with the amplitude and phase as the key features (Kilicarslan et al., 2013; Ogunnika et al., 2008; Cho et al., 2020). Current classifiers used include LSTM for hand grip force prediction Xu et al. (2023), SVM for classification of 7 hand gestures (including rest) (Cho et al., 2020), and Random Forests (RF) and Light Gradient Boosting Machine (LightGBM) to classify 4 hand gestures, including rest (Makeeva et al., 2025).

### 3.6 Optomyography (OMG)

Differences in absorption and reflection of near-infrared (NIR) light in living tissues have been used as a medical diagnostic tool (near-infrared diffuse tomography and near-infrared imaging) (Zhao et al., 2005; Hamaoka et al., 2007). NIR light penetrates the skin and can give information on muscle activity, blood oxygenation, and even hydration. In MMI applications, OMG was first formally proposed by Muhammed and Raghavendra (2015). It is a technique that utilizes NIR light to detect muscle movements by monitoring changes in the reflected light. OMG should not be confused with optical myography (also referred to as OMG), which uses cameras/computer vision to detect the surface deformation that occurs during muscle activities Nissler et al. (2016), Nissler et al. (2015). In other work, such as in McIntosh et al. (2017); Cofer et al. (2022); Maereg et al. (2019); Nsugbe et al. (2020), OMG is referred to as NIR sensing, although the principle is exactly the same. We must also clarify that in Shimabukuro et al. (2025) and Miyake et al. (2024), the term optical myography is used while referring to the NIR case. In this paper we consider the NIR variant (optomyography) as OMG. Near-infrared sensing is a broad term that encompasses a wide range of applications, some of which are not directly related to medical imaging or muscle myography. To maintain clarity, we will use the more specific term optomyography (OMG) in this context, as it more accurately describes the sensing modality relevant to this review.

A constant stream of NIR light is shone at the target area. As muscle activity occurs, the skin and underlying layers deform, causing changes in the amount of light being reflected. NIR light with a wavelength greater than 700 nm can penetrate skin and the changes in the reflected light are measured using photoelectric sensors, such as phototransistor (Muhammed and Jammalamadaka, 2016). OMG is therefore immune to factors such as electromagnetic interference and skin impedance that are highly disruptive in EMG and EIM. In addition, it offers a higher signal-to-noise ratio (Muhammed and Raghavendra, 2015; Muhammed and Jammalamadaka, 2016). However, factors such as skin pigmentation, scarring, moisture, and ambient light noise are limiting factors (Shimabukuro et al., 2025). In particular, ambient light noise must be eliminated or minimized, making OMG especially sensitive to sensor lift-off. OMG only requires an NIR emitter and photoelectric sensor to operate, making the physical arrangement fairly compact. However, the need to block out ambient light often necessitates some bulky cover. OMG is a relatively young technique with good potential applications in prosthetic control (Muhammed and Jammalamadaka, 2016), computer input (Khalikov et al., 2024; Cofer et al., 2022), and rehabilitation/training (Shimabukuro et al., 2025; Miyake et al., 2024). OMG has also been used to detect and monitor facial expressions to assess mental health (Broulidakis et al., 2023; Archer et al., 2023).

#### 3.6.1 Sensor placement

The distance of the emitter-receiver directly affects the amount of light reflected. Therefore, this distance must be optimized based on the wavelength of the NIR being used. For example, in Muhammed and Raghavendra (2015), they found that the optimal focal distance was 6 mm based on the selected wavelength, the photoelectric sensor properties, and the physical layout of the emitter-receiver. In addition, placing the sensors above the areas where skin deformation is largest, such as directly above the active muscles, will produce more observable signals (Shimabukuro et al., 2025). Studies on the effects of sensor placement are currently lacking, and factors such as the influence of limb position effect are yet to be explored.

#### 3.6.2 High density approach

OMG lends itself well to high-density applications because of the minimal components required, just an NIR emitter and receiver. McIntosh et al. (2017) proposed taking advantage of the penetrating NIR by emitting and receiving with all the combinations of emitter and receivers instead of only one emitter-receiver pair. They designed a band with 14 emitter-receiver pairs, and their findings suggest that up to 12 gestures produced discernible patterns. In Khalikov et al. (2024), 50 OMG channels were used to facilitate computer mouse-like controls by decoding 12 hand gestures using a multilayer perceptron algorithm. However, the effects of long-term exposure to NIR light must be studied and carefully considered. HD-OMG applications mean significantly increasing the amount of infrared light the skin is exposed to.

#### 3.6.3 Signal conditioning

OMG requires minimal signal conditioning. After amplification with a differential amplifier, the raw signals can be used Muhammed and Jammalamadaka (2016); Muhammed and Raghavendra (2015). As a result, the sensor lag with OMG is very low.

#### 3.6.4 Feature extraction and classification

The main feature of the OMG signal is the amplitude. Changes in the amplitude are produced by the different ways in which light is reflected from muscle, skin, and subcutaneous layer deformations. These changes appear as peaks and valleys in the signal and by combining the signals from multiple muscles, patterns unique to different gestures can be observed. Currently, OMG classification has been limited to lightweight algorithms such as MLP (Maereg et al., 2019; Khalikov et al., 2024). Few researchers have explored the classification and real-time control using OMG; most studies have been limited to analyzing the OMG signal and not classification.

### 3.7 Lightmyography (LMG)

LMG is a technique that uses light to detect skin deformations produced by muscle activity. In its current rendition, LMG uses both visible and infrared light. Visible light is used to detect skin deformation at the surface, while infrared penetrates the skin layer and provides more information about the deformation of subcutaneous layers, such as the muscles. Therefore, the LMG signal can provide more clarity than OMG. The term LMG was first reported in Shahmohammadi et al. (2021). However, the use of both infrared and visible light was previously reported in Sikora and Paszkiel (2019), where the researchers investigated the effectiveness of using infrared and visible light to detect muscle activity, such as finger movements, using a forearm-mounted sensor. Similar to OMG, a photodetector (phototransistor Guan et al. (2025) or photodiode Shahmohammadi et al., 2021; Shahmohammadi et al., 2023a) is used to detect the changes in reflected light caused by the skin deformation. In Shahmohammadi et al. (2023a), LMG was compared with EMG using the same classifiers, and their findings showed that LMG had higher offline prediction accuracies for 10 classes of hand gestures. In addition, LMG boasts a higher signal-to-noise ratio. LMG has been used for the real-time control of an anthropomorphic prosthetic (Shahmohammadi et al., 2023b) and robotic hands (Godoy et al., 2023a) in addition to estimating the grasp force (Shahmohammadi et al., 2023a). LMG has the same limitations as OMG in that skin conditions, such as pigmentation and scarring, will affect the signal. In addition, measures must be taken to minimize or eliminate ambient light pollution. Fatigue has also been reported to degrade the online performance of LMG controllers (Guan et al., 2025; Godoy et al., 2023a; Shahmohammadi et al., 2023b). However, without further investigation, it is possible that other factors, such as sensor shift, were responsible for the performance drops. The limb position effect and dynamic movements are also not yet understood with LMG.

#### 3.7.1 Sensor placement

To maximize the changes caused by skin deformation, the LED distance from the skin must be optimized. In Shahmohammadi et al. (2023a), they investigated the effect of different LED distances and the use of reflective silicon layers between the skin and LED to maximize the light reflection from skin deformations. The ideal location to place the sensors should be directly above the active muscles. However, LMG is still a young technique, and the exact effects of sensor placement are not yet fully understood. In the current research on LMG, the sensors have been placed arbitrarily around the arm using even spacing. Another key consideration for LMG is sensor fixation. The sensors should be shielded to prevent ambient light interference. Guan et al. (2025) used a neoprene band to completely block out the ambient light. With good ambient light blocking, LMG becomes less affected by sensor lift-off.

#### 3.7.2 High density approach

Similar to OMG, LMG is suitable for high-density applications. In Guan et al. (2025), an LMG band consisting of 5 HD modules was proposed. Each module contained 3 green LED, 2 IR, and 8 phototransistors. With this setup, 10 gestures could be accurately classified offline. The relative simplicity of LMG means that even higher-density constructions could be easily constructed without adding much weight or bulk.

#### 3.7.3 Signal conditioning

LMG signals have a high signal-to-noise ratio, and hence the raw signal can be fed directly into a classifier without any signal conditioning or preprocessing (Shahmohammadi et al., 2021). This means that LMG has a very low lag, especially compared to EMG. Signal amplification to increase the signal-to-noise ratio even further is also considered. Beyond this, there is room to explore other signal conditioning steps. However, current research using LMG has shown good classifier performance on the raw signal (Shahmohammadi et al., 2023a).

#### 3.7.4 Feature extraction and classification

The main feature of the LMG signal is the amplitude. Although LMG is still relatively new, research using LMG has explored using heavyweight classifiers. In Shahmohammadi et al. (2023a) and Godoy et al. (2022b), Temporal Multi-Channel Vision Transformer (TMC ViT) was used to classify LMG and EMG signals, with higher performances reported for LMG. Other algorithms explored include RF, CNN, and BiLSTM (Guan et al., 2025; Shahmohammadi et al., 2023a; Godoy et al., 2022b).

### 3.8 Hybrid/sensor fusion

In our exploration of the various muscle myography techniques, we have shown that each technique has distinct strengths and weaknesses. By combining different techniques (hybrid approach), the clarity of the signals is improved, enabling more useful information to be extracted, such as obtaining joint positions by employing inertial measurement units (IMUs) along with any myography measurement methods. In this section, we discuss practical considerations when implementing hybrid designs and some results from research on hybrid and sensor fusion approaches.

#### 3.8.1 Physical constraints

**FIGURE 4:**
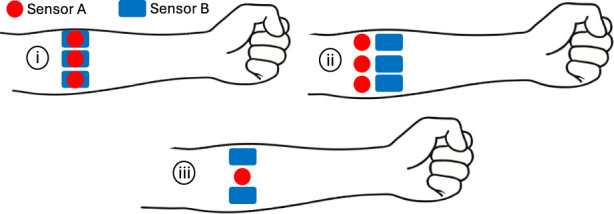
Sensor configurations in hybrid approaches (i) co-located (ii) parallel (iii) alternating.

The propagation of the chosen myography signals dictates the physical design of the sensor-skin interface. For example, EMG requires direct contact with the skin, while OMG and LMG require a direct line-of-sight to the skin. This results in physical constraints that determine how the different sensors can be integrated in a hybrid design. Figure 4 illustrates different possible sensor configurations. Section 4.1 gives a detailed description of the relationship between signal propagation and sensor-skin interface. Consequently, when designing a hybrid MMI, the main considerations are as follows: (1) What are the size requirements? (2) What is the primary myography technique?

When size is the main consideration, choosing techniques with sensors that can be co-located (i.e., targeting the same area) is preferred as illustrated in Figure 4i. In this regard, MMG (especially using IMUs) is an excellent choice. It can be easily integrated with any of the myography techniques without significantly increasing the weight or size of each module. In most cases, FMG techniques use direct skin contact to detect the pressure waves. However, mechanical structures can be used to transmit the pressure waves, freeing space below the sensor. This technique has been used to create hybrids of FMG with EMG and EIM (Briko et al., 2021b). EMG and EIM require direct skin contact, although only the electrodes need to be placed over the target area, resulting in very compact modules. OMG and LMG require a direct line-of-sight to the skin, meaning the area under the sensors cannot be occupied by other sensors. Additionally, they also require a gap (5−6 mm) from the skin and ambient light blocking, making the modules protrude far from the skin. In cases where OMG or LMG are desired in hybrid designs, an alternating sensor arrangement on the forearm axis would provide the most compact design (see Figure 4iii). This method is not commonly used in the literature since only one type of sensor would be at each location/muscle. Alternatively, a parallel arrangement, as in Andreas et al. (2025), is functionally similar to wearing two separate MMI armbands, taking up considerable space (see Figure 4ii).

When the goal is to hybridize a primary technique, considerations center on its physical constraints. As an example, we will consider the case of EMG as the primary technique. Since EMG signals are feature-rich with the added advantage of compact modules, it is often chosen as the primary technique. EMG modules require direct contact with the skin, preventing co-located sensor designs with techniques such as OMG and LMG that require direct access to the skin. FMG and EIM also require direct contact with the skin, seemingly preventing co-located hybrid designs with EMG. In the case of FMG, this problem is solved by using mechanical structures to transmit the pressure waves as discussed previously. With EIM, this was solved by cleverly arranging the EMG and EIM electrodes in a noninterfering pattern. This example demonstrates how the choice of primary technique determines the physical constraints, thus affecting the choice of complementary modalities. The following section outlines various hybrid design approaches that are presently being investigated.

#### 3.8.2 Examples from current studies

EMG signals capture a wealth of data, but suffer from reliability issues due to problems such as noise and crosstalk. Hybrid approaches based on EMG are therefore popular. Andreas et al. (2024) used a combination of 6 sEMG and 6 FMG sensors complemented by 6 IMUs. Their approach was aimed at improving the reliability of pattern recognition. One of the key contributions of their work was to use sensor co-location to reduce the surface area of their device. Briko et al. (2021b) also used sensor co-location with a combination of EMG, FMG, and EIM. Using 2 hybrid sensor channels, they classified 4 wrist gestures. While it may seem difficult to use EIM and EMG due to possible interference, the frequency range of EIM is outside that measured by EMG. Ngo et al. (2022) also used EMG and EIM simultaneously. Their findings suggest that when used this way, EIM can detect muscle force/torque, complementing the EMG signal. Ke et al. (2020) used a hybrid of EMG and FMG to improve the reliability of prosthetic control. They exploited the sensitivity of EMG and the robustness of FMG to classify 3 hand movements using 4 hybrid sensor channels. (Sanford et al., 2017) also investigated EMG and FMG hybrid for prosthetic control, using 4 hybrid sensor channels.

As previously discussed, MMG is good at picking up transients such as the beginning or end of muscle movements, while FMG can provide stable readings during dynamic activities. Consequently, a hybrid of MMG and FMG is a logical choice. In Moqadam et al. (2021), a hybrid of MMG and FMG was implemented to improve pattern recognition in prosthetic control. This combination allowed them to detect finger motions and grasping actions simultaneously.

Cofer et al. (2022) used OMG plus an IMU to detect hand gestures and position in addition to a touch input surface for use in VR applications. The device was used to facilitate 5 input gestures, such as ‘zoom’, for computer control. Ding et al. (2018) proposed a hybrid of sEMG, MMG, and OMG to explore the relationship between grip force and blood oxygenation, in addition to investigating muscle fatigue based on the 3 signals. In their work OMG was not used to monitor the muscle movement; instead the penetrating nature of the NIR was used to monitor blood oxygen metabolism (NIR spectroscopy).

Although the hybrid approach is expected to yield superior outcomes based on intuition, we observed deficiencies in the testing of such devices. For instance, fewer gestures are tested than with the individual approaches. It is thus difficult to assess how much of an improvement can be realized from the hybrid approach. In addition, the combination of muscle myography techniques to use has not been quantitatively studied. Another thing to consider is the possibility of high-density hybrid approaches. Newer techniques like LMG and OMG could also have good performance in hybrid setups.

In this section, we have explored the diverse muscle myography techniques used to detect muscle activity by measuring their physical manifestations. Combining different techniques (hybrid) or using sensor fusion offers a pathway to greater signal clarity and robustness. Furthermore, machine learning has unlocked enhanced functionality in various HMI, assistive, and medical applications, which we explore later on in Section 5.

## 4 Decision framework

This section presents an in-depth exploration of signal propagation and sensor-skin interfaces. We analyze the various signal propagation mechanisms and their implications for sensor design and performance. This is followed by a comparative cross-modality evaluation, examining the strengths and limitations of each technique. Finally, we discuss the most suitable use cases for the different sensing methods, revealing the key trade-offs to consider when selecting an appropriate approach.

### 4.1 Signal propagation and sensor-skin interface

**FIGURE 5:**
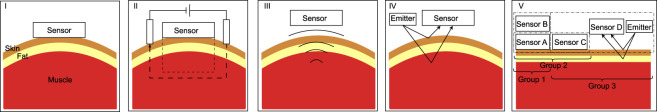
Signal propagation and sensor-skin interfaces (i) Direct skin contact with passive sensing - EMG and FMG (ii) Direct skin contact with active sensing - EIM (iii) Skin-sensor separation with passive sensing - MMG (iv) Skin-sensor separation with active sensing - OMG and LMG (v) Hybrid Combinations. Co-location - group 1, adjacent/alternating - groups 2 and 3.

Sensors fall into two main categories, namely passive and active. Passive sensors directly measure the physical phenomena without emitting any stimulus/signal. EMG, FMG, and MMG fall into this category. Active sensors measure the changes in an emitted signal caused by interacting with the physical phenomena. EIM, OMG, and LMG belong to this category. As a result, setups using passive sensors consume less power. Additionally, the propagation of the muscle myography signal from the muscle → skin → sensor dictates the nature of the skin-sensor interface. In this regard, either direct skin contact or sensor-skin separation is required. Figure 5 summarizes the various sensor types and skin-sensor interfaces. Here, we use the term sensor to refer to the component that measures/picks up the signal (e.g., electrodes in EMG and EIM or photodetectors in OMG and LMG). Techniques such as EMG, EIM, and FMG require direct skin contact for myography signals to propagate to the sensors. For MMG, the signals (vibrations) propagate from the skin into the air gap between the sensor and skin in the case of microphone-based sensors, while IMU based sensors can have contact mediated by the sensor housing. In the cases of OMG and LMG, light travels through the sensor-skin separation before interacting with the skin and subcutaneous layers and being reflected to the sensor. These dynamics of signal propagation and sensor-skin interface dictate the design of muscle myography-based MMIs, determining factors such as power requirements, size, the effect of skin conditions, and suitability for hybrid designs.

### 4.2 Cross-modality comparisons

Tables 1, 2 summarize the cross-modality comparisons for the various muscle myography techniques. Comparisons are made relative to each technique, with ‘very high’ and ‘low’ indicating the highest and lowest relative score between techniques, respectively. Note that these comparisons are relative; thus, ‘low’ when used with a negative connotation simply means relative to the other techniques, it has the worst performance, but is not necessarily bad. These tables illustrate the trade-offs when choosing one technique over another. Complexity in Table 2 refers to the electronic components needed; for example, amplifiers, signal generators, and filters.

**TABLE 1:** Signal characteristics.

Modality	Feature density	Signal clarity	SNR	Preprocessing required	Effect of skin conditions	Long-term stability
EMG	Very high	Low	Low	Very high	Very high	Low - prone to domain shifts
MMG	High	Moderate	Low	Moderate	Low	Very high
FMG	Low	Very high	Very high	Low	Low	Moderate - prone to drift
EIM	Moderate	Moderate-high	Moderate	Moderate	High	Very high
OMG	Moderate	Very high	High	Low	Moderate	Moderate - fluctuates with blood oxygenation
LMG	Moderate	Very high	High	Low	Moderate	Moderate-high
Hybrid	Very high	Very high	Combination dependent	Combination dependent	Combination dependent	Generally higher than its weakest component

**TABLE 2:** Physical specifications.

Modality	Sensor size	Power consumption	Complexity	Suitability for high density	Suitability for hybrid designs
EMG	Very small	Low	High	Very high - however, clinical restrictions apply	High
MMG	Small	Low	High	Low - damping of signals due to increased mass	Very high
FMG	Very small	Low	Low	Highly suitable	Very high
EIM	Very small	Very high	Very high	Not suitable	High
OMG	Large	High	Low	Suitable - however, effect of long term exposure not studied	Low
LMG	Large	High	Low	Suitable	Low

In general, despite the many drawbacks of EMG, its feature-rich signals, compact size, and low power consumption continue to attract researchers since a wide range of applications can be potentially realized. FMG also has similar advantages in size and power consumption, with the added advantages of better signal-to-noise ratio (SNR) and simplicity in terms of circuit design. Yet, the low number of features present in the signals severely limit its potential applications. MMG can be considered to be an all-rounder, with relatively good characteristics across most categories. A major limitation is the difficulty in realizing high-density designs. EIM has several outstanding characteristics; however, its inherent complexity and difficult-to-interpret signals are major limitations, especially in HMI applications. OMG and LMG share many similar advantages and are promising. Reducing the overall size of their sensor modules and conducting more studies on health monitoring applications and skin tone effects are key areas to consider. Finally, as previously discussed, hybrid techniques can combine the strengths of multiple techniques, while mitigating some of their inherent weakness. This area of research is very promising and greatly benefits from the discovery of new techniques, such as OMG and LMG.

### 4.3 What technique to use?

**FIGURE 6:**
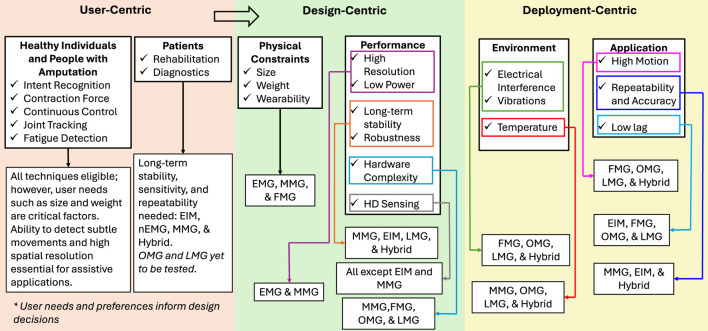
Choosing a technique based on user, design, and deployment-centric approaches.

When choosing a technique, three main approaches can be adopted. The first and most important is the user-centric approach. This takes into account the user’s needs and preferences, such as constraints on size, weight, or battery life. For example, for people with amputation, the size of the stump and the location of the residual muscles are critical and vary between individuals. In principle, meeting the user’s needs should be the main consideration. However, it is a complex and challenging process that is not in the scope of this paper. The second approach is the design-centric approach, where a technique is selected based on achieving predetermined targets, namely physical constraints and performance requirements. This is the most common approach used by researchers. It ensures that the resulting interfaces can compete with state-of-the-art studies on specific performance metrics. The third approach is the deployment-centric approach, where a technique is selected to ensure good performance when deployed in the real world. The anticipated external factors that can cause noise and interference, which degrade the signals, are taken into account. In addition, application-specific metrics are also considered, such as dynamic movements that can generate motion artifacts (e.g., playing games in VR or exercising) or the need for repeatable results (e.g., diagnosing muscle health). Figure 6 illustrates how these different approaches can be used to select a specific technique. In practice, these approaches are used with some overlap and techniques that can satisfy multiple requirements are stronger candidates.

Next, we discuss some of the best use cases for each technique. Although there are many other factors to consider, such as the availability of public data sets and budget, we focus on those related to their inherent strengths and weaknesses. The following section describes the results from different studies where these techniques have been implemented.

1. EMG - is well suited for applications requiring high sensitivity to small or subtle muscle activations and the discrimination of similar gestures, due to its high-bandwidth, feature-rich signals across time, frequency, and time–frequency domains. This makes it particularly effective in prosthetic and exoskeleton control, where residual neural activity can be detected even when muscle contractions are weak or inconsistent. EMG sensors can be easily integrated into compact wearable systems, although the performance is sensitive to electromagnetic interference, electrode placement, skin conditions, and motion artifacts, which can affect long-term stability. Calibration, signal conditioning, and reducing the number of input classes (e.g., gestures) are effective mitigation measures. The feature-rich signals also make EMG valuable in rehabilitation and clinical assessment, where intramuscular or needle EMG (nEMG) may be used to obtain high-fidelity measurements of muscle activity.
2. MMG - is commonly used as a complementary alternative to EMG in HMI applications, providing stable, low-impedance measurements over long durations with simpler sensor setup. While more tolerant to factors such as skin conditions and electrode placement, MMG is more susceptible to motion and external vibration artifacts and typically offers lower spatial resolution than EMG. MMG signals can be acquired from a wider range of locations without strict alignment over specific muscles, enabling flexible sensor placement for wearable and prosthetic systems. Additionally, MMG can capture activity from deeper muscle layers and is particularly well suited for monitoring muscle fatigue and dynamic contractions, making it valuable in rehabilitation and exercise/training applications.
3. FMG - is well-suited for applications where low power consumption, simple hardware, and robust signal acquisition are prioritized over signal richness. FMG provides stable signals that are less sensitive to skin conditions, sweat, and electromagnetic interference, making it particularly effective for long-term wearable use. Although typically offering lower signal richness and spatial resolution than the other techniques, FMG can still support the reliable classification of discrete and moderately complex gestures with relatively low computational overhead. These characteristics make it ideal for applications emphasizing robustness, wearability, and ease of integration, such as exoskeleton control (e.g., gesture selection and proportional control) and sports or rehabilitation monitoring (e.g., tracking muscle engagement and exertion).
4. EIM - has strong long-term stability, meaning the same action produces repeatable patterns even after long periods have elapsed. This makes EIM highly suitable for medical and rehabilitation applications where small deviations from the norm can be detected early. In these use cases, the drawbacks of complexity and power consumption are minimal, since portability requirements are more lenient compared to fully wearable applications.
5. OMG - best suited for applications requiring stability and accurate tracking of specific muscles with minimal computational overhead, low lag, and robustness to skin conditions, motion artifacts, and electromagnetic interference. Consequently, it is highly beneficial in rehabilitation and daily life assistance. OMG also has potential in health monitoring applications, such as fatigue detection and detecting changes in blood oxygenation. OMG’s strengths are also transferable to other applications, such as prosthetic control and teleoperation.
6. LMG - offers low lag control with good responsiveness and stable signals suitable for applications with dynamic or fast actions that could generate motion artifacts for techniques such as EMG and MMG. Examples include computer control, teleoperation, VR, and AR. LMG has also shown comparable performance to EMG-based systems, while using fewer modules and no feature extraction (raw signals). This makes it suitable for systems where computational overheads are vital, such as stand-alone devices. LMG’s potential in rehabilitation and health monitoring applications is yet to be explored; however, some inferences can be drawn from OMG since they share similarities.
7. Hybrid - improved robustness, accuracy, and generalization compared to single-modality approaches. Rather than simply increasing the number of features, hybrid systems enable more reliable decoding by compensating for the limitations of individual sensors (e.g., EMG sensitivity to electromagnetic noise, FMG drift, MMG susceptibility to vibration). While these systems introduce increased hardware complexity, power consumption, and computational requirements, careful design and sensor selection can mitigate these constraints in wearable applications. Hybrid approaches are particularly well-suited to applications requiring high reliability and adaptability, such as VR/AR interaction and teleoperation, where improved signal quality and resilience to environmental and physiological variability outweigh added system complexity. Hybrid approaches are also effective in rehabilitation and exoskeleton control, where multi-modal data provides more useful information, such as tracking joint angles in addition to muscle activations, to achieve harmonious coordination.

## 5 Applications

**FIGURE 7:**
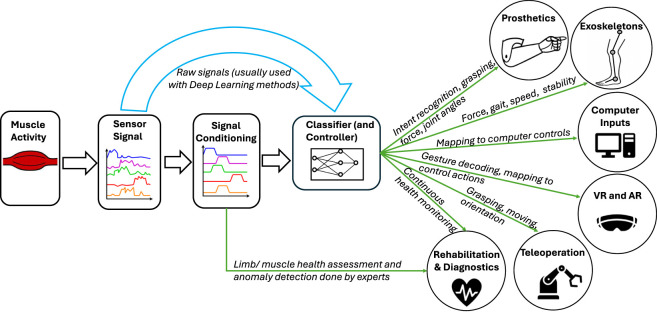
Summary of muscle myography applications covered in this paper.

In this section, we explore the application of muscle myography-based MMIs such as computer input, teleoperation, VR and AR, and prosthetic and exoskeleton control. Although the focus of this review is on MMIs, we also look at the applications of muscle myography to rehabilitation and medical diagnostics. These applications can avail new uses of muscle myography signals, such as continuous health monitoring, which can be implemented on top of the typical uses, such as gesture recognition. Figure 7 summarizes the different applications covered in this section.

### 5.1 Muscle machine interfaces

#### 5.1.1 Computer input

Traditional computer interfaces, such as keyboards, mice, microphones, and touchscreens, are highly effective; however, these interfaces are not suitable for applications where hands-free control or hands-busy control is required and voice input is not feasible. These include applications such as industrial environments, surgery, and unique environments such as space. In addition, assistive applications where users have conditions that prevent them from using traditional computer interfaces effectively. In these cases, muscle activations can be decoded from muscle myography signals, and mapped to specific computer inputs. This application is also known as the Muscle-Computer Interface. In Saponas et al. (2009), hands-free and hands-busy finger gestures were decoded from EMG signals and used to control a portable music player. An HD-OMG approach was proposed in Khalikov et al. (2024) to map 12 hand gestures into computer mouse-like controls. While user adaptation has enabled individuals with disabilities to interact with computers, physical constraints such as button and screen size are difficult to overcome (Kotzé, 2004). Solutions such as creating custom keyboards or touch interfaces partially address this problem, but implementation challenges persist. For example, the physical mounting of a custom keyboard to a mobile platform, such as a wheelchair. BCIs have dominated this area of research; however, muscle myography-based interfaces can also solve all these problems while providing an intuitive and low-profile implementation. For example, Choi and Kim (2007) used EMG to control the movement and clicking of a cursor for individuals with upper arm disabilities.

#### 5.1.2 Teleoperation, virtual reality and augmented reality

The virtual environment enables testing of various experimental setups or prototypes without requiring any hardware implementation. As a result, it can be used in applications such as remote rehabilitation therapy, prosthetics training, and specialized applications such as performing robot-assisted surgery or training remote operators. Teleoperation allows the control of robotic platforms in unsafe environments, such as space or hazardous areas, in addition to applications such as controlling surgical robotic systems (Nguyen et al., 2023). Commonly used input devices include hand-held controllers, vision-based motion tracking, motion capture gloves, and eye tracking, in addition to typical computer interfaces, such as keyboards and mice. To achieve greater user immersion when controlling humanoid avatars or dexterous manipulators, intuitive grasp controls are required. Handheld controllers are not intuitive, in addition to occupying the hands and preventing their use for other concurrent tasks. To solve this problem, motion capture gloves and vision-based tracking have been implemented. However, motion capture gloves have limitations in terms of ergonomics when mass-produced (Sim et al., 2021) and reliability problems during grasp control Bonfert et al. (2023), while vision-based motion capture faces problems such as occlusion (Palombo et al., 2024). Achieving low latency, accurate, and reliable control is critical for improving user control and immersion. Muscle myography-based interfaces could be a potent solution. Their successful implementation in prosthetic and exoskeleton control, explored later, is encouraging for this application. Consequently, research interest in this application is growing. Some current examples include the decoding of finger and grip forces using EMG and mapping them to corresponding forces in VR (Zhang et al., 2022; Shin and Lee, 2024). In Munoz-Novoa et al. (2025), the signal from 8 EMG channels was decoded and used to play games in VR as a rehabilitation activity for individuals with severe strokes or upper limb disabilities. Their study demonstrated the feasibility of using muscle myography, specifically EMG, to mediate VR game control. Although research in this application is still limited, the application of different muscle myography techniques holds great potential and deserves more investigation.

#### 5.1.3 Prosthetic control

Upper limb amputees, such as transhumeral and transradial, retain the ability to control the residual muscles in the stump (Jarrassé et al., 2018; Li and Kuiken, 2009). This means that muscle myography signals similar to healthy limbs are produced and can be used for control. Consequently, prosthetic control is one of the most explored applications of muscle myography-based controllers, offering a promising alternative to traditional myoelectric control. Muscle myography techniques can provide more intuitive control while also enabling the control of more degrees of freedom with low cognitive load. In addition, they offer an alternative to invasive control schemes such as implanted electrodes (Roche et al., 2023) or central interfaces (Del Valle and Navarro, 2013) that require surgery but offer much greater potential, including restoring sensory feedback. Invasive techniques promise to completely integrate a prosthetic limb into the nervous system. However, the associated costs will remain a barrier for most amputees. Muscle myography-based MMIs provide an accessible solution.

EMG is the most applied technique in this application. The ability of EMG to capture a large number of features simultaneously means that it can potentially facilitate seamless control. However, the reliability of EMG is a major drawback. EIM, MMG, FMG, OMG, and LMG have all been applied to prosthetic control. The most popular application is grasp/gesture classification. Here, the intended grasp/gesture of the user is predicted from the muscle myography signal and then applied to the prosthetic hand. This enables the user to switch between different grasps quickly, without the need to reprogram the hand or press any buttons (mode switching), as is typical with traditional control methods. However, issues such as timing and aiming the grasp are not well addressed. In addition, most researchers focused on sequential control, switching between the different grasps and other controls sequentially, rather than simultaneous and proportional control, which is more suitable for daily life applications. Schmalfuss et al. (2018) used EMG to simultaneously control wrist rotation and opening/closing (2 degrees of freedom) of the hand in healthy participants and an amputee. In Muceli and Farina (2011), HD-EMG was used to simultaneously and proportionally control four degrees of freedom. Although their system was only tested with healthy participants, and had lower accuracy than sequential control.

#### 5.1.4 Exoskeleton control

When used for strength augmentation with healthy individuals, an exoskeleton should ideally provide the required movement and force seamlessly without disrupting user movements (Caulcrick et al., 2021). In other words, it should be a natural extension of the body. Achieving this requires intuitive interfacing. In this application, the exoskeleton is used to help the user safely perform strenuous activities (Missiroli et al., 2022) or enhance their abilities; for example, helping a nurse move patients (Yoshimitsu and Yamamoto, 2004) or improving endurance when walking while carrying a load (Nagaraja et al., 2025; Park et al., 2022). The use of BCI based on EEG signals has been explored since the intended actions of the user can be decoded and applied to the exoskeleton with low latency, high accuracy, and low cognitive load (Kilicarslan et al., 2013; Gordleeva et al., 2020), in both healthy and disabled individuals. However, as we have discussed previously, BCI faces many challenges, such as noise and interference, which greatly limit its effectiveness. Alternatively, muscle myography-based MMIs can offer the same benefits while being more stable. The muscle activations of the user are decoded, and the appropriate motion and force augmentation are provided. In particular, the hybrid approach has produced good results and is a promising solution to exoskeleton control (Caulcrick et al., 2021). Exoskeletons have also been used in rehabilitation applications such as gait assistance and hand exercises. In this application, the control requirements are similar to those in the healthy case, and muscle myography-based MMIs can also be used to monitor the recovery rate concurrently, as discussed in more detail in the following sections.

Exoskeletons can also be used to support daily life activities of individuals with disabilities or neuromuscular disorders that reduce their mobility, strength, and endurance (Lee et al., 2024; Bützer et al., 2021; Gerez et al., 2020). In this application, muscle myography-based MMIs can use signals from the muscles of the affected limb to control the exoskeleton. In addition, specific gestures or muscle contractions can be used to access additional controls such as on/off or changing the speed/strength output. In cases where the user has little to no control over their muscles, muscle myography techniques have to be modified to target nontraditional muscles. For example, in Mialland et al. (2022) and Guo et al. (2025), MMG and EMG were used to characterize swallowing motion by measuring muscle activity related to the tongue. This technique could be used as an alternative to EEG and the previously discussed tongue control devices that require some intraoral components to control exoskeletons in users with acute disabilities. In addition, EEG can be complemented with muscle myography signals, such as EMG, improving accuracy and stability (Chowdhury et al., 2017; Kawase et al., 2017).

### 5.2 Rehabilitation and medical diagnostics

The ability of muscle myography signals to capture multiple features related to muscle activity makes them useful for applications beyond MMIs. In fact, techniques such as EMG, EIM, and OMG find their origins in medical applications. Captured features such as contraction levels, joint torques, forces, and speed are useful in assessing limb health. These can be used to check the progress and effectiveness of any treatments or therapies used in rehabilitation. Furthermore, by studying the patterns of the signals, it is also possible to detect the onset of neuromuscular disorders and diseases, enabling early detection and treatment. In rehabilitation applications, the muscle myography sensors used are similar to their MMI counterparts. However, in medical applications, there are significant differences characterized by the need for accurate measurements, in contrast to MMI applications, where the wearability of the device is often the primary concern. As a result, medical sensors provide signals with greater clarity, but cannot be used for wearable applications. Needle EMG (nEMG) is one such example. It offers a much more stable signal compared to sEMG, but requires a needle to probe the targeted muscle (Rubin, 2019; Merletti and Parker, 2004).

In Lee (2022), EMG was used to train stroke patients on how to pedal effectively during rehabilitation. Their findings suggest that by using EMG signals to control the peddling speed, they could significantly improve the balance, gait, and overall muscle control. Beyond exercise, EMG-mediated exoskeleton control can significantly improve neuro-biomechanical coupling during rehabilitation, potentially accelerating recovery and reducing the burden on physiotherapists. Durandau et al. (2022) explored the use of EMG and joint torque sensor fusion to develop subject-specific “neuro-mechanical control model-based control” of an ankle exoskeleton that achieved greater harmonious control compared to the non-assisted case. Gao et al. (2026) investigated the use of EMG-controlled lower-limb exoskeletons for assisted walking in stroke patients. Their results demonstrated improvements in motor coordination and reduced cognitive load, owing to neural adaptation. In Shimabukuro et al. (2025), OMG signals of novices and experts in physiotherapy were compared. Differences in muscle activation could be clearly observed in the signals. The findings of these researchers show that muscle myography signals can be studied in long-term use cases to identify useful trends. For example, this technique could be applied to train in VR and focus on specific weaknesses before any real-world training. This could be useful in training drivers, athletes, surgeons, physiotherapists, and nurses, as example use cases. Blood oxygenation levels change during muscle activity, and NIR light has been used as a detection tool. In Allart et al. (2012), this was used to identify patients with a specific muscular disorder. Visible light reflection can also be used to determine the heartbeat and respiration by measuring the slight changes in light reflection that they produce (Renevey et al., 2018; Abuella and Ekin, 2019). Some commercially available smartwatches already use this technique. OMG and LMG devices could be modified to record these vital signs in addition to muscle activations. EIM has been used to diagnose the progression of muscle disorders such as neurogenic disorders (Rutkove et al., 2002, Rutkove et al. 2010). In assistive applications, muscle myography-based MMIs can be used for daily life assistance, such as controlling a supernumerary arm, while the signal is also remotely analyzed by medical experts to assess disease progression or regression and make any necessary intervention.

Although the use of muscle myography-based HMIs holds great promise for transformative applications in human-computer interaction, the path to widespread real-world implementation remains hindered by various technical, practical, and user-centric challenges, which we discuss in the following section.

## 6 Challenges

We have already explored the detection principles of muscle myography, the different techniques used to record the signals, and their applications in various contexts. However, numerous limiting factors hinder the performance of muscle myography-based MMIs. In this section, we explore the challenges that impact the effectiveness and robustness of the different muscle myography techniques. We also describe mitigation measures that have been recommended or implemented.

### 6.1 Model deployment

**FIGURE 8:**
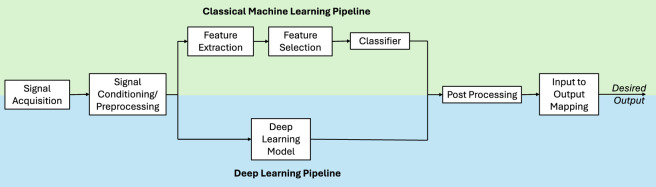
Deployment pipelines for classical machine learning vs. deep learning.

Real-world deployment poses numerous challenges for muscle myography-based systems; factors such as computational overhead, power consumption, and calibration should be considered. Yet, most research concludes after data collection, model training, and offline testing. Progressing to real-world or online deployment represents the true test for the proposed pipelines. Here, we discuss the differences and trade-offs between the two main deployment pipelines: machine learning (ML) and deep learning (DL). Figure 8 shows the deployment pipelines for the two approaches.

As discussed previously, signal conditioning involves steps such as amplification and filtering. Post-processing involves steps such as setting the confidence thresholds for the predictions, setting the output frequency, and the number of consecutive identical predictions needed to trigger an output change (output smoothing). Input-to-output mapping is the process of binding the model’s predictions to specific outputs/actions. For example, mapping hand gestures to computer input, such as scrolling or clicking. Another example is to map muscle activation patterns to predictions on limb health.

ML classifiers generally offer very low latency predictions, while DL models (especially heavyweight models) have a heavier inference step. However, ML approaches require manual feature extraction and selection. Feature selection is typically performed during the training phase only. This process can be time-consuming and may not yield the best feature set (Shen et al., 2019). During inference time, only the features selected in the training phase are extracted. This feature extraction step also contributes delays to the overall latency. However, it is important to note that these contributions in latency are minimal and the biggest contributor to the overall latency is the window size which is not an inherent attribute of either pipeline. DL requires minimal to no feature extraction, as the models can learn high-level abstractions from raw data (Gopal et al., 2022; Shen et al., 2019). Consequently, during the deployment phase, DL pipelines offer higher accuracy predictions (Aarotale and Rattani, 2024). However, this comes at the cost of increased computational overhead, which hinders deployment on edge devices. For heavyweight models a server may be required to house the model and do all the processing. Quantization can significantly reduce the size of the models; however, this typically comes at the cost of reduced accuracy (Alajlan and Ibrahim, 2022). Light-weight ML models are easier to deploy on edge devices and can achieve low latency predictions with very low power consumption (Gomez-Bautista et al., 2025; Vitale et al., 2022); however, their performance on large gesture sets (greater than 6) is significantly lower. This is one of the reasons why antagonistic control of prosthetics remains the preferred approach, even with modern prosthetics. ML models also offer better interpretability, meaning that is easier to diagnose problems and implement fixes such as repositioning sensors, recalibration, or tuning. DL models function more like black-boxes making it difficult to interpret what is happening. As a result, troubleshooting and debugging are more difficult, which affects the reliability. This is especially important in safety-critical applications such as exoskeleton and prosthetic control, as well as applications requiring reliability, such as diagnostics and rehabilitation. Evidently, choosing a pipeline is a complex problem that would benefit from more research involving online testing. The current trend has been to choose heavyweight DL models that achieve higher offline prediction accuracies on large gesture sets.

### 6.2 The limb position effect

The limb position effect is a phenomenon where the inter-class differences between different gestures/movements are reduced based on the position of the limb, for example, the outstretched arm versus the bent elbow (Scheme et al., 2010; Radmand et al., 2014). Different limb positions may require the use of some muscles for stabilization (Fougner et al., 2011), resulting in some muscles contracting before any gesture is made. This results in higher classification errors and poor online performances of the classifiers (Geng et al., 2017). Most data for the classification of muscle myography signals is collected from subjects in a seated or resting position, especially in EMG research (Scheme et al., 2010; Fougner et al., 2011). This data does not reflect the dynamic and unpredictable nature of daily life activities. In studies on the limb position effect in sEMG (Scheme et al., 2010; Fougner et al., 2011; Radmand et al., 2014), it was found that some limb positions could increase classification errors by over three times. While the limb position effect has been explored mainly in the context of sEMG, it also affects the remaining muscle myography techniques. However, the extent needs to be investigated as some techniques may be less affected.

Simply including more data from different limb positions in the training can significantly mitigate the limb position effect (Scheme et al., 2010; Samuel et al., 2017; Amsüss et al., 2013a). This means that a lot more data has to be collected from each subject, lengthening the data collection process and possibly inducing fatigue. As a result, while this approach is the most logical, its implementation is not as straightforward. Another approach is to include additional sensors, such as IMU or accelerometers, to detect the limb position and then include this data in the training set. In Geng et al. (2012) and Geng et al. (2017), a cascade classification approach was attempted. IMU data was used to classify the limb position, which was then fed to the next classifier together with the EMG data. This resulted in a decrease in the classification error; however, the online accuracy remained lower than the offline accuracy. The limb position effect must be resolved to improve the reliability of MMIs in daily life applications. While studies on the limb position effect have focused on EMG, it affects all other muscle myography techniques, yet its effects have not been considered in most research.

### 6.3 Motion artifacts

Motion artifacts are noise or distortions in the sensor signal caused by movement that is not related to the targeted muscle activity. Motion artifacts are produced by cable sway, sensor shift/lift-off, changes in sensor fixation due to dynamic activity, and mechanical forces such as vibration, impact, and bending of components (Schlink et al., 2020; Roland, 2020). It is difficult to eliminate motion artifacts; therefore, filtering is the most common approach, especially in EMG research. Other measures include optimizing the sensor fixation or using flexible sensors (Roland, 2020; Radmand et al., 2016). The problem of motion artifacts is mainly reported in EMG research; however, it also affects other techniques and needs to be mitigated to improve the reliability of the MMIs. The high-density approach has also been used to alleviate motion artifacts in EMG (Schlink et al., 2020; Stango et al., 2014) and FMG Wu et al. (2020). Since most research is done in highly constrained environments, the problem of motion artifacts is not well observed. Testing during dynamic real-world scenarios is the best way to investigate the effect of motion artifacts on classifier/controller performance and test the effectiveness of any mitigation measures.

### 6.4 Sensor shift and lift off

**FIGURE 9:**
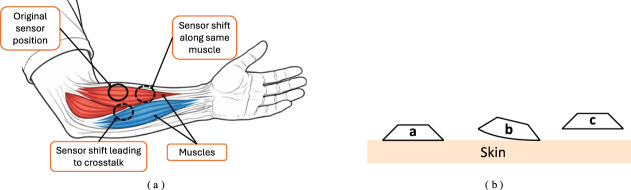
Comprehensive overview of sensor positioning and contact states. **(a)** Differentsensorshiftscenarios. **(b)** (a) Fullcontact (b) Partialsensorlift-off (c) Fullsensorlift-off.

Sensor shift occurs when a sensor is moved from its original or intended position. This can result in crosstalk, reducing the clarity of the signals, and in some cases, sensor shift can lead to dead channels where there are no changes in the sensor readings under all conditions. Figure 9a illustrates different sensor shift scenarios. The sensor shift problem affects all different muscle myography techniques; however, EMG is particularly susceptible to sensor shift. Sensor lift-off occurs when the sensor loses partial or complete contact with skin, as shown in Figure 9b. This often results in dead channels that do not respond to muscle activity.

Sensor shift and lift-off distort the overall readings from the device, degrading the classifier/controller performance. Daily life activities are highly dynamic and unpredictable, and often result in sensor shift and/or lift-off. As a result, MMIs are often unreliable in daily use applications. Sensor fixation is critical to prevent or mitigate these problems; however, if the fixation is too tight, it can lead to the dynamic coupling of muscles, resulting in crosstalk (Orizio, 1993). High-density approaches are less affected by sensor shift and lift off. Having a large number of sensor channels means the effect of a few dead channels or crosstalk is diluted (Pan et al., 2015; Stango et al., 2014).

### 6.5 Skin conditions

The skin surface mediates the connection between sensors and the muscles they are targeting. Consequently, changes in skin conditions can have adverse effects on the signal. For example, skin hydration affects its impedance, impacting EMG and EIM. Well-hydrated skin has lower impedance compared to dry skin. While physical activity generates sweat, which reduces impedance. Factors such as temperature, humidity, physical activity, and even the choice of lotion affect the skin hydration (Camilion et al., 2022; Iizaka, 2017; Ryosuke et al., 2021).

The physical condition of the skin itself is also critical. The presence of scars and abrasions is detrimental to all muscle myography techniques. Another factor to consider is skin pigmentation that affects OMG and LMG. The color of the skin determines the wavelengths of light reflected. This means that the device may have to be recalibrated based on the skin color of the user, limiting the potential use of these devices. One solution is to use a colored medium between the skin and sensor, ensuring consistency (Shahmohammadi et al., 2021, Shahmohammadi et al., 2023a).

### 6.6 Clinical acceptability

To ensure that muscle myography-based MMIs are safe for daily use, they must meet relevant clinical guidelines. The clinical acceptability of EMG requires that the number of electrodes be limited to 8. However, many research works (especially HD-EMG) ignore this. Findings from these works suggest that increasing the number of electrodes can improve the signal clarity and minimize the effects of noise, motion artifacts, sensor shift, and lift-off, significantly improving the results (Stango et al., 2014; Kok et al., 2024; Pan et al., 2015; Simon et al., 2024). Clinical acceptability in EIM means that the high-density approach cannot be realized because of the increase in the amount of current that is injected into the skin. With OMG and LMG, the wavelength and intensity of infrared and near-infrared light used must be within clinically acceptable ranges. Focusing on only the performance metrics without considering clinical and ergonomic requirements prevents much of the proposed research from progressing beyond the research sphere. This is particularly evident in the fields of prostheses, rehabilitation, and assistive devices, where few proposed works have impacted the people they are designed for.

### 6.7 Physiological and psychological factors

The amplitude and intensity of muscle contractions is affected by both physiological (Mukund and Subramaniam, 2020; Allen et al., 1992) and psychological factors (Amagliani et al., 2010; Hidaka et al., 2004). This is reflected in the muscle myography signals. When the training data is collected in highly constrained lab environments, the resulting classifiers/controllers will not be robust. In Amsüss et al. (2013b) and Kaufmann et al. (2010), the long-term stability of EMG classifiers was tested. In both papers, the performance of the classifiers diminished with each passing day. Factors such as electrode shift and lift-off were eliminated by placing the sensors in the same position each time. Ultimately, the exact reason could not be identified. However, some possible causes included the decline in subject motivation throughout the experiments. User fatigue also could not be ruled out as a cause. Guan et al. (2025) also observed a sharp decline in classifier performance when performing online tests for LMG-based control. They attributed this decline to possible user fatigue. The effects of the physiological and psychological states of the user on real-time performance need to be studied further to build reliable classifiers suitable for daily life use. Other solutions could include using a recalibration scheme to update the classifier parameters each time. Additionally, differences in muscle tone and skeletal structures between individuals also affect the muscle myography signal, sensor placement, and fixation. Godoy et al. (2023b) investigated the use of transfer and few-shot learning as methods to reduce the effect of fatigue, sensor shift, and musculoskeletal differences on classifier performance when using sEMG. As users become more accustomed to the system over time through increased usage, their adaptation can help mitigate the impact of some reliability challenges. However, there is a lack of long-term use studies to investigate the role of user adaptation for the different muscle myography techniques.

### 6.8 Transition from functional domain to activity domain

**FIGURE 10:**
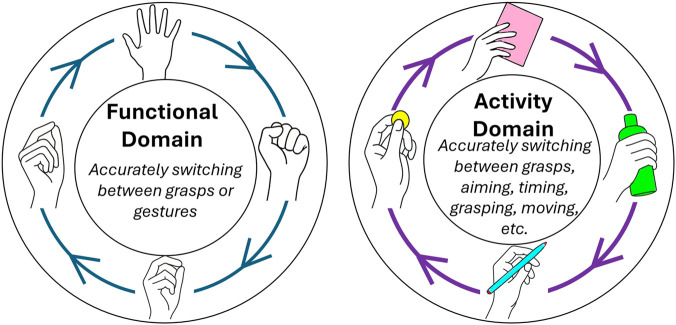
Differences between the functional and activity domains. The activity domain is highly varied and dynamic, while the functional domain is fairly controlled.

When developing and testing MMIs, researchers often rely on data collected in constrained lab environments, a problem that we have mentioned numerous times throughout this paper. In addition, when the proposed systems are deployed, they are mostly tested in the functional domain, where the controller’s ability to switch between different functions accurately is the main testing criterion. For example, in prosthetic applications, this would mean testing if the controller correctly switches to the intended or predicted grasp. However, these devices are intended to be used in the activity domain where completing tasks involves much more than switching between functions. For example, in prosthetic applications, factors such as hand orientation, timing, motion, and simultaneous control of additional degrees of freedom, such as the elbow, are essential for performing tasks. The activity domain imposes the challenges previously discussed. This disparity between the two domains is shown in Figure 10. Consequently, good performance in the functional domain does not imply good performance in the activity domain. Fougner et al. (2011) proposes that testing should be done in the activity domain during clinical or lab trials. This means more tests need to be performed, requiring greater user participation and lengthening the research process. However, a true representation of the expected performances of the proposed system can be obtained, and implementation challenges/solutions will be exposed, enabling further research.

This section has underscored the numerous challenges that continue to hinder the widespread adoption of muscle myography-based human-machine interfaces. While we have also presented the various mitigation measures explored by researchers, the successful elimination or substantial alleviation of these challenges remains a key priority for future work in this rapidly evolving field. The emergence of datasets other than EMG, such as MMG Abbas et al. (2025), FMG Zakia and Menon (2022), is also a significant step forward.

## 7 Conclusion

Traditional input devices such as keyboards, touchscreens, and joysticks remain effective for many tasks, but they impose constraints: they occupy the hands, limit accessibility for users with disabilities, and struggle to deliver truly embodied interaction in immersive environments. Muscle myography-based interfaces address these gaps by enabling direct, non-invasive decoding of muscle activity, offering a pathway to more natural, intuitive, and inclusive interaction.

This review has outlined the landscape of muscle myography techniques and their role in shaping the next-generation of human–machine interfaces. Beyond their technical promise, these modalities challenge long-standing assumptions about interaction by moving away from hand-centric, device-bound input toward more embodied, muscle-driven control. Such a shift has profound implications for accessibility, assistive technologies, and emerging domains like immersive interactions with virtual and augmented reality.

Yet, the path from laboratory prototypes to everyday adoption remains constrained by issues of robustness, usability, and user diversity. Variability in physiology, limb position effects, motion artifacts, and sensor reliability all highlight the need for systems that adapt not only to signals but also to people and contexts. Hybrid sensing, multimodal integration, and machine learning offer partial solutions, but the deeper challenge lies in designing interfaces that remain intuitive and resilient in the messy reality of daily life.

For the HMI community, this creates fertile ground for exploration: how can myography-based interfaces be designed to support long-term use, adapt to individual users, and balance performance with comfort and acceptability? How can such systems expand beyond assistive applications to enable novel forms of interaction in virtual and physical spaces? Addressing these questions will require interdisciplinary collaboration across engineering, design, and human factors.

Muscle myography is still an emerging area within assistive technologies and human-machine interfaces, but it offers a rare opportunity: to create interfaces that are easy to use and intuitive by facilitating embodied interactions. By bridging physiology and interaction design, future research can move toward interfaces that feel less like tools to be mastered and more like natural extensions of the body.
